# The impact of dyslipidemia on skeletal health - from an immunoregulatory perspective

**DOI:** 10.3389/fimmu.2026.1774535

**Published:** 2026-03-11

**Authors:** Baisong Zhou, Shuai Li, Jiyu Song

**Affiliations:** 1 Changchun Institute of Biological Products Co., Ltd., Changchun, China; 2 Hospital of Stomatology, Jilin University, Changchun, China

**Keywords:** bone homeostasis, dyslipidemia, immune regulation, lipid metabolism, osteoporosis

## Abstract

Dyslipidemia and obesity are key risk factors for cardiometabolic diseases and are also linked to osteoporosis and other bone disorders. Evidence shows lipid metabolism influences bone homeostasis largely through immune regulation. This review first explains how abnormal lipid metabolism disrupts adipogenic and osteogenic differentiation in bone marrow mesenchymal stem cells and alters adipokines like leptin and adiponectin, upsetting bone formation and resorption and leading to bone loss. It then examines the lipid–immune–bone axis. In innate immunity, high lipid levels shift macrophages from M2 to pro-inflammatory M1, increase bone-resorbing cytokines such as TNF-*α* and IL-1*β*, and trigger neutrophil senescence and lipid peroxidation with excess reactive oxygen species, all of which promote osteoclast formation and suppress bone growth. In adaptive immunity, hyperlipidemia changes T-cell metabolism, weakens Treg function, and drives Th17 differentiation; this Th17/Treg imbalance boosts osteoclasts via RANKL, IL-17, and related pathways. Meanwhile, in inflammation, B cells switch from producing OPG to releasing RANKL and G-CSF, while Breg-derived IL-10, IL-35, and TGF-*β*1 protect bone. The review also highlights how M1 macrophages and Th17 cells work together to worsen bone damage. Understanding these immune mechanisms could lead to new treatments for metabolic bone diseases. Despite these advances, the translation of these preclinical findings into clinical practice remains a challenge that warrants further investigation.

## 1 Introduction

Dyslipidemia is a metabolic disorder characterized by quantitatively or qualitatively abnormal circulating lipid levels--clinically defined by elevated total cholesterol, triglycerides, and low-density lipoprotein cholesterol (LDL-C), alongside reduced high-density lipoprotein cholesterol (HDL-C). It is now well established as a significant risk factor for chronic noncommunicable diseases, particularly cardiovascular diseases (CVD), including coronary artery disease (CAD) and ischemic stroke, as well as type 2 diabetes mellitus (1). Given its high global prevalence and strong causal links to morbidity and mortality, dyslipidemia represents a pressing public health challenge.

Chronic diseases characterized by dyslipidemia or lipid metabolism disorders are often accompanied by immune imbalance and chronic inflammation. Elevated endogenous cholesterol levels can promote the activation of various pro-inflammatory immune cells in both the innate and adaptive immune systems. In animal models of metabolic syndrome, dietary cholesterol loading can lead to significant accumulation of macrophages in adipose tissue and arterial walls, thereby inducing local inflammation and promoting systemic chronic inflammatory responses (2, 3). Additionally, *in vitro* studies have shown that mast cells can promote low-density lipoprotein uptake through their granules upon stimulation, which in turn induces cholesterol accumulation in macrophages (4, 5). In antigen-specific autoimmune Th1 cells, enrichment of membrane cholesterol can bias T cells towards an inflammatory phenotype (6). Overall, the interaction between abnormal cholesterol metabolism and immune cells plays a key role in the occurrence and progression of related diseases.

Triglycerides are transported in the blood in the form of very low-density lipoproteins (VLDL) and chylomicrons, which are typical triglyceride-rich lipoproteins. Triglyceride-rich lipoproteins and their remnant particles not only carry residual cholesterol but also exert direct pro-inflammatory effects on macrophages (7). Elevated triglycerides can exacerbate local and systemic inflammatory responses by inducing phosphorylation of JNK and p38 MAPK in macrophages, inhibiting cholesterol efflux, promoting macrophage death and foam cell formation (8). Moreover, elevated triglycerides may also promote the development of CVD such as atherosclerosis by reshaping the pro-inflammatory transcriptional profile of T cells (9). After being hydrolyzed by lipoprotein lipase (LPL), triglyceride-rich lipoproteins generate a large amount of free fatty acids (FFA), among which unsaturated fatty acids can be metabolized through strictly regulated and highly specific oxidation pathways to produce potent pro-inflammatory or pro-resolving lipid mediators. These fatty acid oxidation derivatives have received considerable attention in recent years as they have been shown to significantly affect the functions of various immune cells, especially antigen-presenting cells and T cells, and thus play important immunoregulatory roles (10). Overall, the abnormal levels of plasma triglycerides and related lipids and their interactions with immune cells play a key role in the occurrence and progression of multiple chronic diseases.

**Figure 1:**
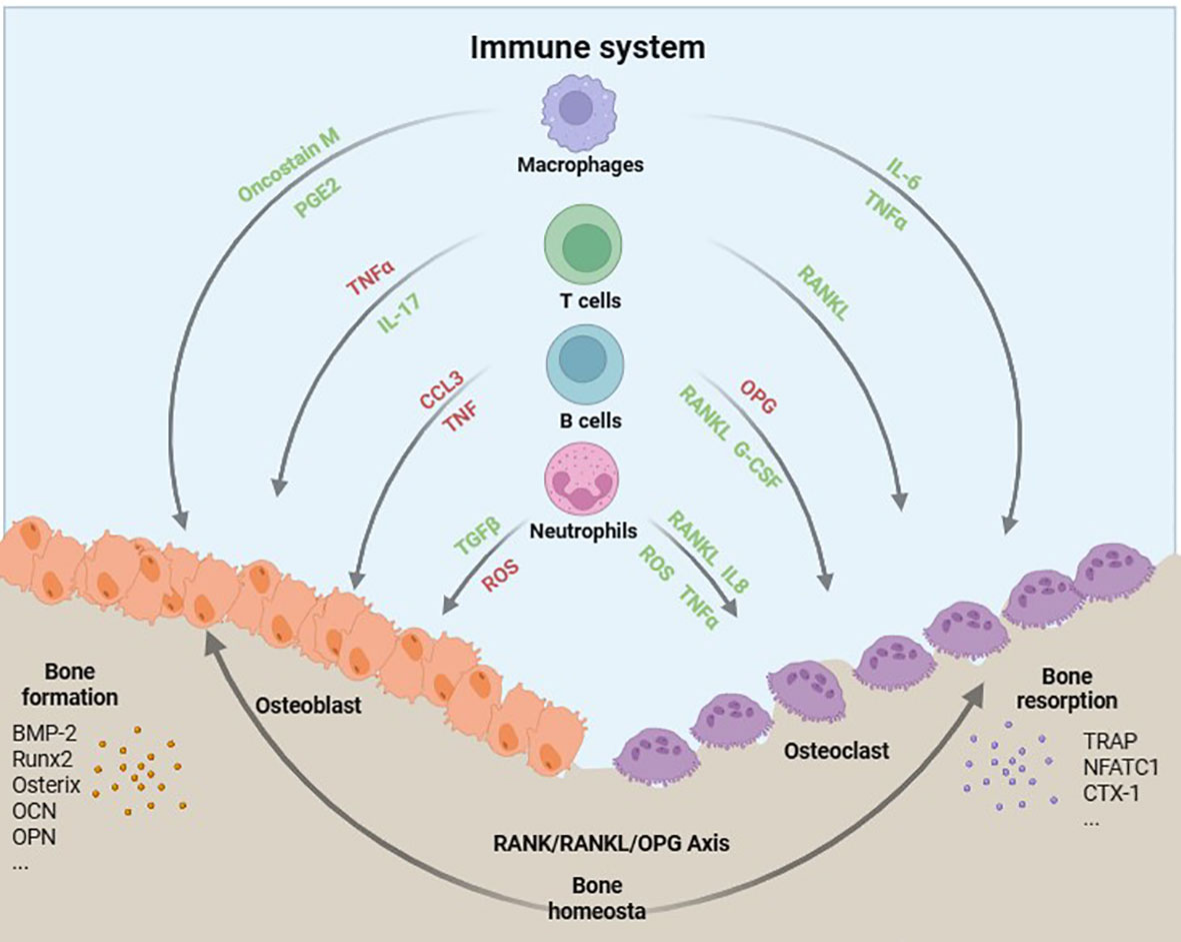
Schematic illustration of immune cell interactions with bone cells.

In recent years, emerging evidence has demonstrated the pivotal role of lipid metabolism in the regulation of bone homeostasis. It has been observed that disturbances in lipid metabolism can disrupt the delicate equilibrium between bone formation and resorption, thereby contributing to the development of bone disorders such as osteoporosis (11–13). However, the precise mechanisms underlying the impact of lipid metabolism on bone homeostasis remain incompletely elucidated. Recent investigations have unveiled intricate interplays between lipid metabolism and immune function within the context of skeletal health. The immune system, specifically immune cells and inflammatory mediators, play a crucial role in the regulation of bone remodeling and maintenance (**Figure 1**) (14, 15). One potential mechanism through which lipid metabolism influences bone homeostasis is by modulating immune function. Dysregulation of lipid metabolism can result in chronic low-grade inflammation, activation of immune cells, and induction of the release of various inflammatory and immune factors, including cytokines and chemokines (16). These factors have the potential to directly impact bone cell function by promoting bone resorption and inhibiting bone formation (13, 16). The comprehensive understanding of the intricate interplay among lipid metabolism, immune function, and bone homeostasis is crucial for the development of preventive and therapeutic strategies targeting bone diseases. By specifically targeting lipid metabolism and immunomodulation, it may be feasible to restore the delicate equilibrium of bone remodeling processes and enhance overall skeletal health.

In this review, we will explore the current understanding of how lipid metabolism modulates immune function and subsequently impacts bone homeostasis. By elucidating these mechanisms, our aim is to contribute to the advancement of knowledge in this field and provide insights into novel strategies for maintaining optimal bone health in individuals with metabolic disorders.

## 2 The disruption of bone homeostasis resulting from aberrant lipid metabolism

Bone serves not only as a mechanical organ but also plays a crucial role in metabolism and various endocrine functions (17). Bone metabolism encompasses the dynamic processes of osteoblast-mediated bone formation and osteoclast-driven bone resorption within the skeletal tissue. Furthermore, A clinical cross-sectional study conducted in Chinese individuals demonstrated that participants with higher body fat percentages exhibited a significantly elevated risk of osteoporosis and non-vertebral fractures, independent of body weight, physical activity levels, and age (18). Another clinical study investigating the associations between lipid profiles, body mass index (BMI), and bone mineral density (BMD) in postmenopausal women revealed that serum lipid parameters were closely correlated with osteoporosis. Notably, total cholesterol emerged as an independent risk factor for osteoporosis in this population subgroup (19). The evidence presented the strong correlation between lipid metabolism and bone metabolism.

Adipokines, bioactive mediators secreted by adipocytes, play a pivotal role in maintaining bone homeostasis. Since osteoblasts and adipocytes originate from a common pool of bone marrow mesenchymal stem cells (BMSCs), their differentiation pathways often exhibit a competitive dynamic. Specifically, a reciprocal relationship typically characterizes osteogenesis and adipogenesis in the bone marrow (20). This inverse correlation is largely driven by the competition between lineage-specific transcription factors; for instance, Peroxisome proliferator-activated receptor gamma (PPARγ), a master regulator of adipogenesis, can inhibit osteogenic signaling (13). The administration of the PPARγ agonist rosiglitazone to mice can effectively suppress the expression of osteoblast transcription factors Runx2, osterix, etc., thereby promoting the differentiation of BMSCs into adipocytes and inhibiting osteogenesis (12). C-fos plays a crucial role in osteoclastogenesis, and activation of PPARγ induces upregulation of c-fos expression, leading to osteoclast activation and subsequent bone resorption. Leptin, a protein hormone secreted by adipocytes and primarily synthesized in white adipose tissue, is recognized as a crucial regulator of bone metabolism. Mice lacking the gene responsible for leptin production display diminished bone mass and impaired bone formation (21, 22). Whereas, the administration of leptin treatment leads to a reduction in bone marrow adipose tissue and an increase in bone formation in ob/ob mice deficient in leptin (23, 24). Additionally, leptin exerts a positive influence on bone health by suppressing osteoclastogenesis through inhibiting the expression of RANKL (25). Based on a cohort of 1,167 postmenopausal women and a 25-year follow-up interview conducted in Japan, the findings of an epidemiological study revealed that low levels of leptin were identified as a significant independent risk factor for the development of long bone fractures (26).

The polypeptide or protein Adiponectin (ADPN) is an endogenous substance secreted by adipocytes, exhibiting significant biological activity. It demonstrates highly specific expression in differentiated adipocytes and can be found abundantly in plasma (27, 28). Scholars have discovered that ADPN facilitates the proliferation of osteoblasts, resulting in an elevation of alkaline phosphatase (ALP) activity, as well as increased production of osteocalcin, type I collagen, and mineralized matrix. Additionally, it stimulates both the proliferation and differentiation of human osteoblasts (29–31). The action of ADPN also involves the inhibition of osteoclastogenesis through its ability to suppress nuclear factor κB (NF-κB) and p38 signaling pathways (32). However, there is currently no consensus in the field of ADPN research. Studies have demonstrated that ADPN exerts a dual effect depending on its location and mode of action: it directly impacts osteoblasts by inhibiting their proliferation, while in the central signaling pathway, it promotes osteoblast proliferation (33). Similarly, Mitsui et al. (34) observed a significant increase in both bone mass and bone formation in a mouse model with adiponectin overexpression compared to wild-type mice. However, Ealey et al. (35) reported contrasting findings, demonstrating that mice with adiponectin overexpression exhibited lower bone mineral content in the femur and reduced peak load capacity in the femoral neck compared to control mice. Furthermore, several studies have revealed an increase in bone mass in mouse models with knockout of the adiponectin coding gene (36–38). Therefore, further investigations are still required to explore the effects of adiponectin on bone metabolism and its underlying mechanisms.

## 3 The modulation of lipid metabolism affects immune function, thus impacting bone homeostasis

### 3.1 The influence of lipid metabolism on innate immunity

#### 3.1.1 Macrophages

Macrophages play a crucial role in the innate immune system, being responsible for maintaining tissue homeostasis, resolving immune responses during pathogen infections, and facilitating tissue repair and remodeling during periods of tissue damage (39, 40). One of the key attributes of macrophages isage polarization refers to the immunological characteristics by these cells, and their function plays a pivotal role in maintaining bone health (41). The macrophages can be classified into two distinct categories: M1 and M2 (42). M1 macrophages are primarily characterized as inflammatory cells that possess the ability to eliminate invading pathogens and actively participate in a diverse range of antigen presentation processes (43). Notably, the secretion of cytokines such as tumor necrosis factor alpha (TNF-*α*), interleukin (IL)-6, and IL-1*β* along with the production of reactive oxygen species (ROS) and nitric oxide (NO) are prominent features associated with M1 macrophages (44). Meanwhile, the progressive accumulation of pro-inflammatory cytokines generated by M1 macrophages ultimately leads to enhanced bone resorption and heightened osteoclast activity. On the contrary, M2 macrophages exhibit anti-inflammatory properties by primarily producing TGF-*β* and IL-10 (45). They actively participate in tissue repair and immune resolution, while concurrently inhibiting bone resorption and promoting osteogenesis. The M1/M2 classification framework provides a basis for macrophage categorization; however, it is increasingly evident that macrophages possess a dynamic nature enabling them to continuously adapt in response to prevailing conditions. In recent years, metabolic activity has emerged as a pivotal regulator of macrophage activation, function, and biology (46). Disruption in lipid metabolism can significantly impact macrophage functionality. The consumption of a high-fat diet and the presence of obesity trigger inflammatory responses, resulting in the activation and excessive accumulation of macrophages (47, 48). The subsequent content will delve into elucidating the intricate relationship between lipid metabolism, macrophages function and bone health.

Chemical substances involved in lipid metabolism possess the capacity to influence macrophage polarization, thereby regulating bone health. Fatty acids constitute the primary constituents of lipid synthesis, and elevated levels of fatty acid intake can induce M1-type polarization in macrophages, leading to an augmentation in the production of pro-inflammatory factors (49). Chronic inflammation is one of the causes of osteoporosis. As an important regulator of inflammatory response, TNF-*α* exists in trimeric form as a product of activated macrophages, mast cells, and natural killer cells (50). Among them, M1 macrophages are the main cells that produce macrophage-derived TNF-*α*. TNF-*α* has an important effect on bone differentiation. On one hand, TNF-*α* stimulates osteoclast differentiation through NF-κB, upregulating multiple target genes such as RANK (16). On the other hand, it inhibits osteoblast differentiation by suppressing bone-forming factors like Runx2 (51). As a result, the balance between bone resorption and formation is disrupted, leading to the occurrence of osteoporosis. Recent clinical data have also confirmed that patients with osteoporosis exhibit elevated levels of serum TNF-*α* (52), indicating the potential role of TNF-*α* as a crucial factor in the improvement of osteoporosis. Moreover, IL-1*β* is predominantly secreted by M1 macrophages and exerts regulatory effects on M-CSF production, osteoclastogenesis stimulation, inhibition of osteoclast apoptosis, and suppression of bone cell vitality through activation of the NF-κB/RANKL signaling pathway (53, 54). Furthermore, M1 macrophages not only secrete cytokines but also function as precursors for osteoclasts, serving as a reservoir for these bone-resorbing cells (31). Hence, it is reasonable to hypothesize that an increase in M1 macrophages could potentially exacerbate bone resorption.

The M2 macrophages, in contrast, are characterized by an anti-inflammatory phenotype (55). Lipid ketone bodies generated through *β*-oxidation of long-chain fatty acids have the ability to polarize macrophages towards the M2 subtype and enhance the production of anti-inflammatory factors. A notable example is ursodeoxycholic acid, a bile acid, which induces M2 macrophages polarization in ob/ob mice and subsequently mitigates inflammation resulting from excessive fat accumulation (56). Cholesterol plays a crucial in lipid metabolism. Excessive accumulation of unesterified cholesterol in macrophages can trigger calcium transients and subsequently lead to macrophage death (57). The administration of Nim enhances SIRT1 expression in RAW 264.7 cells, thereby promoting cholesterol homeostasis reprogramming and subsequently increasing the proportion of M2 macrophages (58). This highlights the intricate association between lipid metabolism and M2 macrophage polarization. It is noteworthy that M2 macrophages are intricately involved in the regulation of bone homeostasis. Cytokines associated with M2 macrophages, such as IL-4 and IL-13, exert an inhibitory effect on bone resorption by suppressing the differentiation of osteoclasts and reducing the activity of mature osteoclasts through RANKL/RANK/OPG system (59). M2 cytokines can also suppress the expression of osteoclast marker factors, including RANK and tartrate-resistant alkaline phosphatase (TRAP), thereby impeding the differentiation and activation of osteoclasts. Moreover, recent studies have emphasized the role of M2 macrophages in promoting osteogenesis. The interactions among macrophages, preosteoblasts, and mesenchymal stem cells (MSCs) were demonstrated by the upregulation of osteogenic markers and enhanced bone mineralization in MSCs co-cultured with M2 macrophages (14, 15). In summary, the above supports that lipid metabolism regulates bone differentiation by regulating macrophage polarization.

#### 3.1.2 Neutrophils

Skeletal aging is characterized by a state of low bone turnover and the accumulation of adipose tissue in the bone marrow. Neutrophils are considered to play a pivotal role in the pathogenesis of chronic inflammatory diseases, and metabolic disorders induced by high-fat diet consumption can contribute to neutrophil senescence (60). The research findings indicated that neutrophils of the pro-inflammatory and senescent subtypes have been observed to accumulate in the bone marrow, where they secrete significant quantities of grancalcin. This protein has the ability to bind to the PLXNB2 receptor and subsequently activate downstream pathways in MSCs within the bone marrow. Consequently, this process ultimately hinders osteogenesis while promoting adipogenesis in stromal cells present in the bone marrow, thereby contributing to the development of OP and fragility-related fractures (61). Furthermore, a high-fat diet can induce lipid peroxidation in neutrophils, leading to heightened generation of ROS such as hydroxyl radicals (62). Hydroxyl radicals can elevate levels of oxidative stress, thereby inducing cellular damage and dysfunction, which in turn exert detrimental effects on skeletal homeostasis. ROS are the main types of free radicals involved in bone remodeling and damage. Elevated levels of ROS within osteoblasts can induce cell cycle arrest at the DNA synthesis phase, thereby reducing their proliferative capacity (63). Additionally, ROS can also inhibit the expression of Runx2 and Osterix, resulting in decreased osteogenic activity (64). Glutathione (GSH) plays a crucial role in regulating levels of ROS, and it enhances osteogenic differentiation by upregulating the expression of collagen I, OCN, and ALP (65). Elevated levels of GSH also exert an inhibitory effect on RANKL-induced osteoclastogenesis through the suppression of the ROS/NF-κB signaling pathway (66). The excessive presence of ROS, however, can impede the efficiency of GSH conversion. The use of H_2_O_2_ is a common approach in constructing oxidative stress models. By reducing the expression of osteogenic markers such as ALP and Collagen I, H_2_O_2_ inhibits mineralization (67). Moreover, bone formation is also affected by H_2_O_2_-induced senescence of BMSCs and adipocyte differentiation (68). H_2_O_2_ is capable of activating the NF-κB and MAPK signaling pathways during osteoclastogenesis (69). Additionally, H_2_O_2_ can suppress autophagy by inhibiting the Nrf2 and mTOR signaling pathways, thereby promoting RANKL-mediated osteoclast differentiation (70). In summary, dysregulation of lipid metabolism disrupts bone homeostasis through neutrophil senescence and oxidative stress.

### 3.2 The influence of lipid metabolism on adaptive immunity

#### 3.2.1 T cell

T cells are a cornerstone of adaptive immunity system, derived from hematopoietic stem cells. They regulate the process of bone remodeling by secreted various cytokines and growth factors (71, 72). T cells are further categorized into Th1, Th2, Th17, and regulatory T cells (Treg) by distinction of their cell surface molecules. These subsets differ in produced cytokines and therefore function (73). Recent studies on the molecular and functional interplay between lipid metabolism and T cell biology have found that lipid metabolism seems to play a key regulatory role in the levels of T cell transcription, epigenetics and post-translational regulation (74). Oxidized lipids significantly enhance the production of RANKL and promote the expression of lectin-like oxidized LDL receptor-1 (LOX-1) by affecting T lymphocyte function, indicating the role of oxidized lipids in immune mediated bone loss (75). Lipid metabolism may regulate the process of bone remodeling by affecting the activation, differentiation and effector functions of various types of T cells, which is a key mechanism of abnormal bone metabolism in hyperlipidemia.

##### 3.2.1.1 Treg cell

Tregs are a subpopulation of T cells and play a crucial role in controlling immune responses (76). Hyperlipidemia profoundly changes the host’s ability to regulate immune responses by affecting Tregs metabolism (77). Functionally, hyperlipidemia-induced changes in Tregs lead to decreased suppressive function, and the production of effector cytokines, including IFN-γ, IL-2 and IL-4. During this process, the activation of Akt2 drive glycolysis in Tregs while decrease the transcription of FoxP3 (necessary for the development and function of Tregs) and alter cytokine gene expression by Tregs (78). This functional change in Tregs caused by hyperlipidemia may directly affect the ability of osteoclastogenesis. On the one hand, Treg cells inhibit osteoclast differentiation from peripheral blood mononuclear cells in a cytokine-dependent, such as TGF-*β*, IL-10 and IL-4 (79, 80). IL-10 and IL-4 are inhibitory cytokines secreted by Treg cells, which can inhibit the differentiation of osteoclasts by upregulating the secretion of osteoprotegerin (OPG) and downregulating the expression of RANKL and M-CSF (81, 82). On the other hand, *in vitro* research results indicate that Treg cells can remove CD80/CD86 from the surface of osteoclast precursors by CTLA-4 mediated trans-endocytosis. This direct contact potentially lead to reduced co-stimulation by osteoclasts, thereby interfere with bone resorption by affecting osteoclast development and function at multiple stages (83). An animal study transferred FoxP3^+^CD4^+^CD25^+^ Treg cells sorted by flow cytometry into lymphocyte-deficient RAG-1^–/–^ mice, and found the transfer of Treg cells resulted in decreased bone resorption, while bone formation remained unaffected. The analyses for other T cell subsets, such as CD4^+^CD25^+^IFNγ^+^ for Th1 cells, CD4^+^CD25^+^IL-4^+^ for Th2 cells, and CD4^+^CD25^+^IL-17^+^ for Th17 cells, were not detectable by flow cytometry, which confirmed Treg cells could indeed regulate bone homeostasis through direct engagement of osteoclast precursors independently of other T cell subsets *in vivo (*84).

##### 3.2.1.2 Th cells

The balance between Tregs and T helper (Th) cells has always been a focus of immunological research. During an immune response, effector Treg cells and Th cells generally play opposite roles with the former suppressing and the latter promoting the immune inflammation (85). Research has shown that hyperlipidemic humans and mice exhibit increased levels of inflammatory cytokines in their serum, and exhibit increased inflammatory T cell response (86–88). Analysis of transplant rejection in ApoE^-/-^ mice (a physiologically relevant model of hyperlipidemia) revealed that hyperlipidemia promotes accelerated rejection of vascularized cardiac allografts by inducing anti‐donor Th17 reactivity and production of IL‐1 (89). Another study on atherogenic LDb mice (had significantly elevated levels of total cholesterol as well as triglyceride) also showed increased serum IL-17, which was associated with promoted polarization and inflammatory function of autoimmune Th17 cell (90). IL-17-producing Th17 is a specialized inflammatory subset, which is identified as an osteoclastogenic Th cell subset that links T cell activation and bone resorption (91, 92). According to recent reports, Th17 cells plays a key role in bone loss in various inflammatory diseases, such as rheumatoid arthritis and periodontal disease (93, 94). Th17 cells have a positive effect on osteoclast generation *in vitro* and they balance the microenvironment and facilitate osteoclast differentiation (92). Th17 cells could potently induce osteoclastogenesis by secreting IL-17 (36, 95), IL-1, IL-6 (96), RANKL (97), and TNF (98). It is worth noting that the inflamed gastrointestinal mucosa of IBD patients is heavily infiltrated by Th17 cells, and Th17 related cytokines are produced in excess (98). The osteoclastogenic function of the Th17 cells may be a key mechanism of bone loss in IBD mouse mode, and it is expected to become a potential therapeutic target (99). IL-17 represents a potent osteoclastogenic cytokine (100), not only because IL-17 can stimulate osteoclast differentiation by inducing the expression of RANKL in osteoblasts and osteocytes (101), but also IL-17 can facilitate local inflammation by recruiting immune cells, resulting in the production of abundant inflammatory cytokines such as TNF-*α*, which further induce osteoclast differentiation (102).

Recent findings suggest that lipid metabolism plays a vital role in the differentiation and function of Th17 cells (74). A High-Fat Diet Promotes Th17 Cell Differentiation and increased the expression of enzymes in the fatty acid biosynthetic process, such as acetyl-CoA carboxylase 1 (ACC1, known as a master kinase involved in fatty acid metabolism (103, 104)). Pharmacological inhibition or gene deletion of ACC1 leads to impaired Th17 cell differentiation, while the overexpression of Acaca (encodes ACC1) induced Th17 cells *in vivo*, resulting in largely unchanged expression of and IL-4 (104). Intriguingly, the inhibition or deletion of ACC1 causes human T cells mouse T cells toward Treg cells at the expense of Th17 cells (105). This transition towards Treg cell differentiation in Acaca-deficient cells can be overcome by extrinsic fatty acid supplementation, indicating that fatty acid metabolism directs the balance between Th17 and Treg cell (103, 104, 106). The balance of Treg/Th17 cell immune homeostasis is crucial for maintaining bone homeostasis. When Th17/Treg cell balance transfer to Th17 cells, aggressive osteoclast differentiation leads to bone resorption related diseases (71, 97).

#### 3.2.2 B lymphocyte

Chan et al. (107) showed that a chronic high-fat diet accelerated age-related loss of bone trabeculae and altered the number of B lymphocyte in the bone marrow and blood. Among other things, B lymphocyte may be the key interface between lipid metabolism and bone metabolism for mutual regulation. The canonical role of B lymphocyte is in antibody production, but they also serve as antigen-presenting cells and respond to innate and adaptive stimuli to produce cytokines. B lymphocytes are characterized by a variety of phenotypes, which are described in terms of B cells and Breg cells.

##### 3.2.2.1 B cells

In the context of atherosclerosis, dyslipidemia affects B cells proportions, phenotypes, and subpopulations. Schmitz et al. (108) used flow cytometry to examine the phenotypic characteristics of circulating B cells in humans, and found that serum high-density lipoprotein (HDL) levels were negatively correlated with the IgD expression of B cells and with naive B cells. Rincón et al. (109) found that atherosclerotic mice had higher levels of B cells activation, as evidenced by their higher surface expression of CD80, CD86, CD40, and CD95, and higher serum levels of IgG, which activates T follicular helper (TFH) cells and induces the expression of inflammatory factors such as interferon gamma (IFN-γ). In addition, abnormal lipid metabolism promotes systemic and vascular inflammatory changes by inducing multiple mediators (IL-6, IL-1, and TNFα) (110, 111). Cholesterol is directly associated with inflammation through activation of the NLRP3 inflammasome, which creates a chronic, low-grade inflammatory environment phenotype (112). B cells under physiological conditions play a key role in osteoclast inhibition through secretion of OPG receptor decoys, but the inflammatory environment allows for altered cytokine expression and immune cell profiles, which can redirect the effects of B cells on bone remodeling to bone resorption. B cells in the inflammatory milieu stimulate osteoclast activation through the secretion of RANKL (113–115), along with the secretion of granulocyte colony-stimulating factor (G-CSF) (116), leading to the proliferation of osteoclast progenitor cells. As key regulators of bone remodeling, B cells communicate with osteoclasts and osteoblasts in an environmentally dependent form with various cytokines (117).Thus the contrasting role of B cells in osteoclastogenesis in the context of environmental changes induced by abnormalities in lipid metabolism supports their critical role in metabolic bone remodeling homeostasis (118, 119).

##### 3.2.2.2 Breg cells

**Figure 2:**
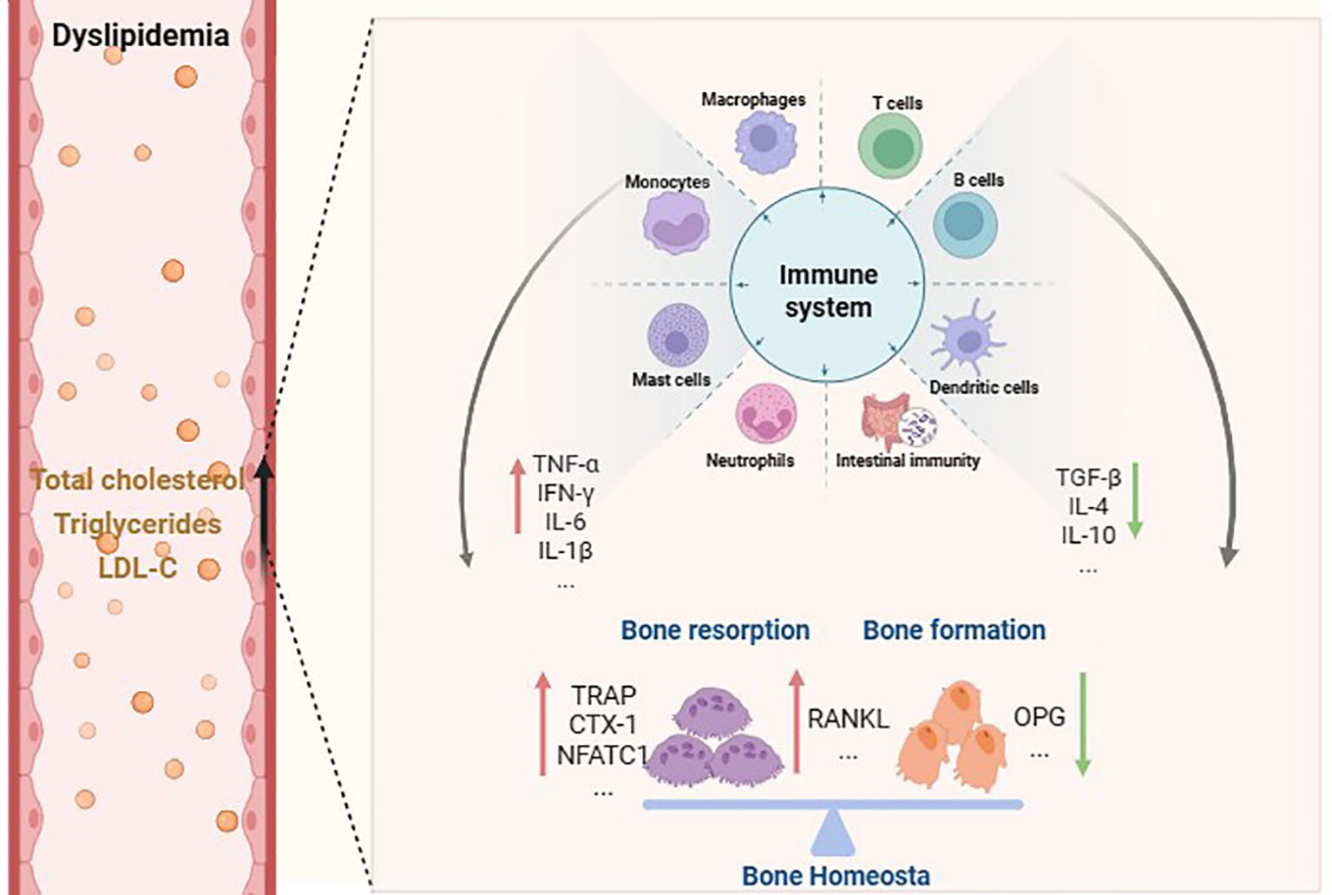
Schematic illustration of the mechanisms underlying immune cell-mediated regulation of bone homeostasis in dyslipidemia.

In addition to traditional antibody-secreting B cells, the immunomodulatory role of B-regulatory lymphocytes (Breg) in lipid metabolism is increasingly being recognized (120). Breg cells are increased in hypercholesterolemic mice and inhibit Th cells by expressing anti-inflammatory factors such as IL-10, IL-35, and TGF-*β*1, suppressing macrophage antigen presentation, and negatively regulating humoral immunity to attenuate the inflammatory response in atherosclerosis and prevent lesion progression (121). Meanwhile, Breg cell-produced cytokines have been demonstrated to influence osteogenic differentiation. IL-10 enhanced osteoblast development in a recent animal model by downregulating miR-7015-5p (122). IL-10 not only promotes osteoblast formation but also inhibits Ca^2+^ mobilization and NFATc1 signaling in osteogenic precursors, inhibiting osteoclast development (123). Through the STAT1/STAT3 signaling pathway, IL-35 promotes Breg cell differentiation (124). The binding of IL-35:IL-35-R appears to suppress osteoclast formation via OPG release and subsequent downregulation of RANKL to reduce osteoclastogenesis (125). TGF-*β*1 and bone morphogenetic protein (BMP) signals through the SMAD or MAPK pathways, resulting in a signaling cascade that upregulates pro-osteoblastic cytokines such as RUNX2 (126, 127). TGF-*β*1, like IL-10, appears to inhibit NFAT signaling and decreases RANK expression on osteoblasts (127). Given the above effects of anti-inflammatory factors, the transfer of Breg cell subsets appears to delay the onset of osteoporosis (128). As shown in **Figure 2**, the potential mechanism of immune cell-mediated bone homeostasis regulation in dyslipidemia.

## 4 Conclusion

The present review highlights the intricate interplay between lipid metabolism and bone metabolism, underscoring the pivotal role of immune regulation in this physiological process. Dyslipidemia reshapes innate and adaptive immunity and, in turn, disrupts skeletal remodeling. Oxidized lipids acting via receptors such as LOX−1 and PLXNB2 promote pro−inflammatory macrophage polarization and enhance osteoclastogenic cytokine production, while altered T−cell metabolism shifts the Th17/Treg balance toward a pro−resorptive profile. These immunometabolic changes converge on canonical bone pathways—including RANKL/OPG, NF−κB, and Wnt/β−catenin—leading to increased osteoclast differentiation, impaired osteoblast and BMSC function, and potential osteocyte dysfunction in the setting of hyperlipidemia. Therefore, it is imperative to conduct further investigation into the immunomodulatory mechanisms underlying the interaction between lipids and bone metabolism, as this knowledge holds significant potential for comprehending and preventing bone-related diseases. The focus of future research efforts should be directed towards elucidating the specific roles of immune regulation in lipid and bone metabolism, as well as exploring novel therapeutic strategies to enhance the prevention and treatment of associated diseases.

## 5 Perspectives on future research

Given the crucial role of immune regulation in bone health among individuals with hyperlipidemia, interventions targeting lipid metabolism and immune signaling pathways hold promise for the prevention or treatment of osteoporosis. However, current translational research in this area remains relatively fragmented, with most evidence still at the preclinical stage. Firstly, traditional lipid-lowering therapy is one of the most accessible intervention methods at present, but clinical data on BMD and fracture outcomes in patients receiving different lipid-lowering regimens are highly heterogeneous, and there is a lack of prospective studies simultaneously assessing immune phenotypes and bone outcomes. Secondly, the development of immunomodulatory therapies targeting key cytokines and signaling pathways in the “lipid-immune-bone axis” is expected to establish a mechanistic bridge between inflammation control and bone protection, providing new intervention ideas for bone damage related to hyperlipidemia. However, to truly translate these potential strategies into clinically feasible bone protection plans, more systematic and comprehensive translational research is still needed.
